# Toward the solution of the protein structure prediction problem

**DOI:** 10.1016/j.jbc.2021.100870

**Published:** 2021-06-11

**Authors:** Robin Pearce, Yang Zhang

**Affiliations:** 1 Department of Computational Medicine and Bioinformatics, University of Michigan, Ann Arbor, Michigan, USA; 2 Department of Biological Chemistry, University of Michigan, Ann Arbor, Michigan, USA

**Keywords:** protein structure prediction, deep learning, template-based modeling;, free modeling, end-to-end structure prediction, multiple sequence alignment, contact map, distance prediction, DCA, direct coupling analysis, FM, template-free modeling, HMM, hidden Markov model, MRF, Markov random field, MSA, multiple sequence alignment, PDB, Protein Data Bank, PSSM, position-specific score matrix, SI, sequence identity, TBM, template-based modeling

## Abstract

Since Anfinsen demonstrated that the information encoded in a protein’s amino acid sequence determines its structure in 1973, solving the protein structure prediction problem has been the Holy Grail of structural biology. The goal of protein structure prediction approaches is to utilize computational modeling to determine the spatial location of every atom in a protein molecule starting from only its amino acid sequence. Depending on whether homologous structures can be found in the Protein Data Bank (PDB), structure prediction methods have been historically categorized as template-based modeling (TBM) or template-free modeling (FM) approaches. Until recently, TBM has been the most reliable approach to predicting protein structures, and in the absence of reliable templates, the modeling accuracy sharply declines. Nevertheless, the results of the most recent community-wide assessment of protein structure prediction experiment (CASP14) have demonstrated that the protein structure prediction problem can be largely solved through the use of end-to-end deep machine learning techniques, where correct folds could be built for nearly all single-domain proteins without using the PDB templates. Critically, the model quality exhibited little correlation with the quality of available template structures, as well as the number of sequence homologs detected for a given target protein. Thus, the implementation of deep-learning techniques has essentially broken through the 50-year-old modeling border between TBM and FM approaches and has made the success of high-resolution structure prediction significantly less dependent on template availability in the PDB library.

Proteins are the macromolecules that are nearly ubiquitously responsible for carrying out the various functions necessary to sustain life, from cell structural support, immune protection, enzymatic catalysis, cell signal transduction to transcription and translation regulation. These diverse functions are made possible by the unique three-dimensional structures adopted by different protein molecules. The landmark study by Anfinsen in the 1970s showed that the tertiary structure of a protein is dependent on its amino acid sequence (1). Since then, understanding the protein sequence–structure–function paradigm has become a cornerstone of modern biomedical studies. Due to significant efforts in genome sequencing over the last 4 decades (2, 3, 4), the number of known nucleotide sequences in the GenBank database (5) has grown to over 2600 million as of 2021. Of these nucleotide sequences, approximately 200 million have been translated into the corresponding amino acid sequences and deposited in UniProt (6). Despite the impressive accumulation of data, the amino acid sequences themselves provide only limited insight into the biological functions of each protein, as these are essentially determined by their three-dimensional structures. An interesting exception to this are intrinsically disordered proteins, which have been estimated to make up roughly 30% of proteins in the human proteome, and may be functional despite lacking well-defined tertiary structures (7). However, even intrinsically disordered proteins may undergo disordered-to-ordered transitions and adopt tertiary structures upon binding to their partners and performing their biological functions (8, 9).

Among the most accurate experimental methods for determining the structures of proteins are X-ray crystallography (10), NMR spectroscopy (11), and cryo-electron microscopy (12). However, due to the significant human effort and expenses required to experimentally solve a protein structure, the growth in the number of solved protein structures has lagged far behind the accumulation of protein sequences. So far, the structures of approximately 0.18 million proteins have been deposited in the Protein Data Bank (13) (PDB), which accounts for less than 0.1% of the total sequences in the UniProt database (14). This percentage was 0.7% in 2010 and 2% in 2004; therefore, it is apparent that the gap between the number of known protein sequences and experimentally solved protein structures is continually widening. Thanks to the tremendous effort made by the community over the last few decades (15, 16, 17, 18, 19, 20, 21, 22, 23, 24, 25, 26, 27, 28, 29), an increasing portion of the genes in organisms have had their tertiary structures reliably modeled by computational approaches (30, 31, 32, 33, 34, 35, 36). In addition, numerous high-quality structural models are being created every day by online structure prediction systems (22, 23, 27, 29, 37, 38, 39, 40, 41, 42, 43), which have been used to assist various biomedical studies, including structure-based protein function annotation (44, 45, 46, 47, 48), mutation analysis (49, 50, 51, 52, 53, 54, 55, 56), ligand screening (57, 58, 59, 60, 61, 62, 63, 64), and drug discovery (65, 66, 67, 68, 69, 70). Thus, the development of high-accuracy protein structure prediction methodologies represents perhaps the most promising, yet challenging, approach to address the disparity between the number of known protein sequences and experimentally solved structures, while also elucidating the fundamental principles that govern the protein sequence-to-structure-to-function paradigm.

Historically, protein structure prediction approaches have been generally categorized as either template-based modeling (TBM) or template-free modeling (FM) methods. TBM methods construct models by copying and refining the structural frameworks of existing proteins, called templates, identified from the PDB, while FM methods predict protein structures without using global template structures. The accuracy of TBM is contingent on the quality of the alignments between the target protein and the identified templates, which is often dependent on the evolutionary distances between the query and templates. For proteins with sequence identities (SI) >50% to the templates, for example, models produced by TBM can have up to 1 Å RMSD from the native structure for the backbone atoms. For proteins with 30 to 50% SI, the models often have ~85% of the core regions within an RMSD of 2 to 5 Å to the native structure. However, when the SI drops <30% (the Twilight Zone) (71), modeling accuracy sharply decreases due to alignment errors and the lack of significant template hits (72, 73, 74). Despite this drop-off in accuracy, in theory, the protein structure prediction problem could be solved using TBM even at a stringent sequence identity cutoff (<25%) if algorithms were able to identify the best templates from the PDB library (75). Nevertheless, this has yet to be achieved in practice due to the difficulty and error in recognizing distantly homologous templates (76).

Unlike TBM methods, FM methods have been traditionally used to model proteins for which no homologous templates can be identified from the PDB library. Since FM methods do not use global template information, they traditionally rely on physics- and/or knowledge-based energy functions and extensive sampling procedures to construct protein structure models and therefore often have been referred to as *ab initio* or *de novo* modeling approaches (21, 23). Due to the inherent inaccuracies associated with these procedures, FM has not historically achieved the same accuracy as TBM. However, recently the field has witnessed a remarkable achievement in that, for the first time, the gap between the TBM and FM accuracies has largely been bridged through the use of deep learning, in particular end-to-end learning, to build protein structure models (27, 28, 77, 78). This strategy resulted in the construction of experimental quality structures by the top performing group, AlphaFold2 (77), for approximately 35% of proteins that lacked significant homologous templates in the PDB and 77% of proteins with homologous templates in the most recent community-wide blind test of protein structure prediction approaches, compared with an average of 0% and 20%, respectively, in the previous three assessment rounds (79, 80, 81, 82). In this review, we will start with an overview of the history of protein structure prediction, followed by a discussion of the recent progress and challenges covering the state of the art of the field. In particular, we will highlight the profound impact brought about by deep learning, where the breakthrough in end-to-end learning has largely solved the single-domain protein structure prediction problem (83).

As a supplemental aid, Table 1 lists links to the discussed methods so that readers may access these useful resources, and Figure 1 provides an overview of the important achievements and milestones over the last 50 years that are covered in this review. The selection of the lists can be subjective and limited by the space of the article.

**Table 1:** List of the useful methods for protein structure prediction covered in this review with available links to access the resources

Multiple sequence alignment (MSA) construction	
PSI-BLAST	https://blast.ncbi.nlm.nih.gov/Blast.cgi
HHBlits	Web server- https://toolkit.tuebingen.mpg.de/tools/hhblits Downloadable version -https://github.com/soedinglab/hh-suite
Jackhmmer	https://www.ebi.ac.uk/Tools/hmmer/search/jackhmmer
Hmmsearch	https://www.ebi.ac.uk/Tools/hmmer/search/hmmsearch
DeepMSA	https://zhanglab.ccmb.med.umich.edu/DeepMSA/
Threading and Fold-recognition	
LOMETS	https://zhanglab.dcmb.med.umich.edu/LOMETS/
HHsearch	https://github.com/soedinglab/hh-suite
MUSTER	https://zhanglab.dcmb.med.umich.edu/MUSTER/
map_align	https://github.com/sokrypton/map_align
EigenTHREADER	https://github.com/psipred/eigenthreader
CEthreader	https://zhanglab.dcmb.med.umich.edu/CEthreader/
DisCovER	https://github.com/Bhattacharya-Lab/DisCovER
RaptorX	http://raptorx.uchicago.edu
Full-length Structure Assembly for Template-Based Modeling (TBM)	
I-TASSER	https://zhanglab.dcmb.med.umich.edu/I-TASSER/
MODELLER	https://salilab.org/modeller/
RosettaCM	https://www.rosettacommons.org/software/license-and-download
SWISS-MODEL	https://swissmodel.expasy.org/
Phyre2	http://www.sbg.bio.ic.ac.uk/phyre2/
Fragment Assembly Simulation Methods for Free Modeling (FM)	
Rosetta	Web server: https://robetta.bakerlab.org Downloadable version: https://www.rosettacommons.org/software/license-and-download
QUARK	https://zhanglab.dcmb.med.umich.edu/QUARK/
FragFold	https://github.com/psipred/fragfold
Co-evolution and Deep Learning-Based Contact/Distance Prediction	
PSICOV	http://bioinfadmin.cs.ucl.ac.uk/downloads/PSICOV/
CCMpred	https://github.com/soedinglab/CCMpred
GREMLIN	http://gremlin.bakerlab.org
NeBcon	https://zhanglab.dcmb.med.umich.edu/NeBcon/
MetaPSICOV	http://bioinf.cs.ucl.ac.uk/psipred/
ResPRE	https://zhanglab.dcmb.med.umich.edu/ResPRE/
TripletRes	https://zhanglab.ccmb.med.umich.edu/TripletRes/
RaptorX-Contact	http://raptorx.uchicago.edu/ContactMap/
MSA Transformer	https://github.com/facebookresearch/esm
Deep Learning-Based Full-length Structure Prediction	
AlphaFold	https://github.com/deepmind/
D-I-TASSER	https://zhanglab.dcmb.med.umich.edu/D-I-TASSER/
D-QUARK	https://zhanglab.dcmb.med.umich.edu/D-QUARK/
trRosetta	https://yanglab.nankai.edu.cn/trRosetta/
DMPfold	Web server - http://bioinf.cs.ucl.ac.uk/psipred/ Downloadable version https://github.com/psipred/DMPfold

**Figure 1:**
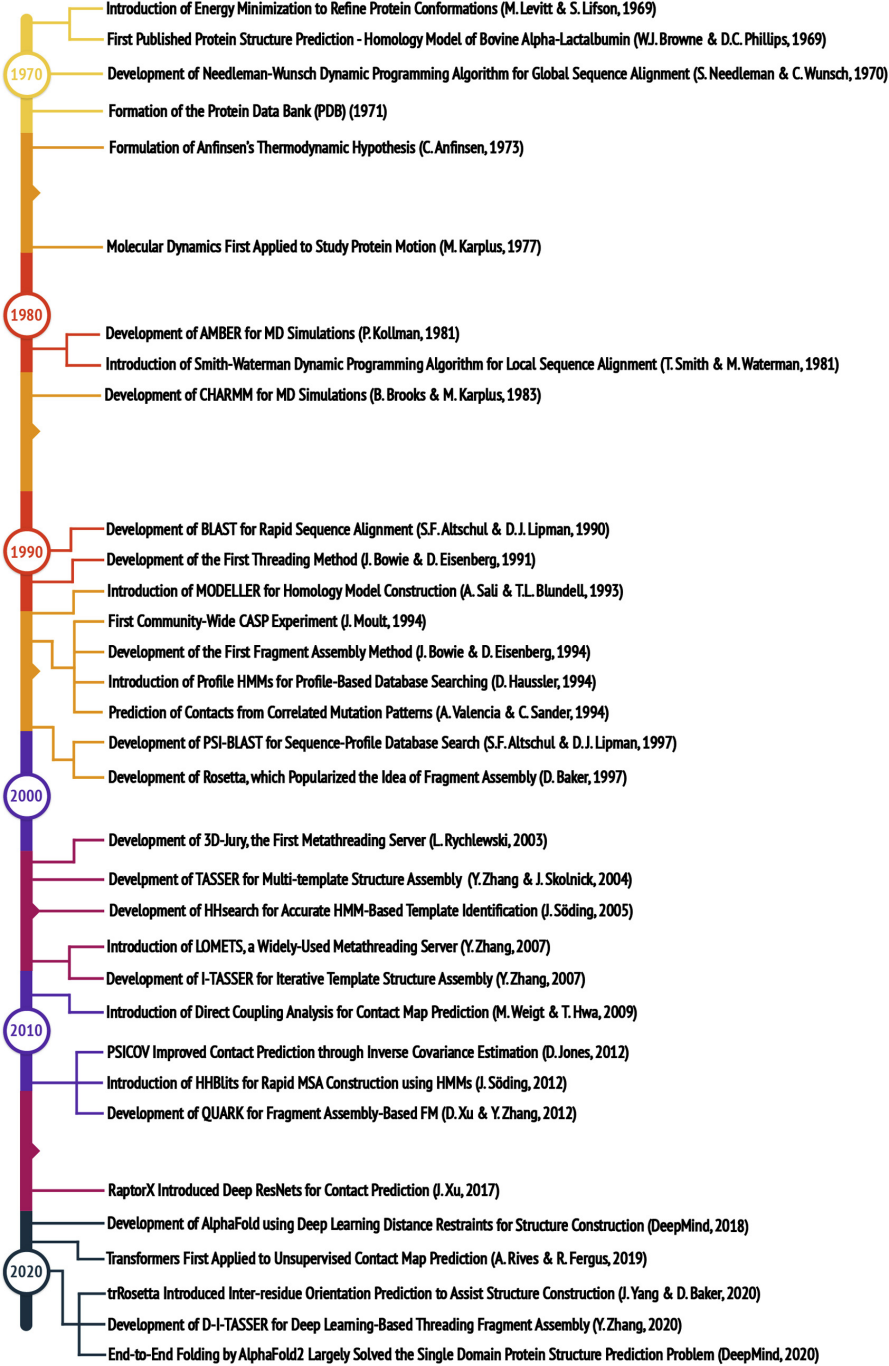
**Important milestones in protein structure prediction that are covered in this review**.

## An overview of the history of protein structure prediction

### TBM—homology modeling

The first published attempt at TBM, and protein structure prediction in general, can be traced back to 1969 when Browne *et al.* (84) built a model for bovine alpha-lactalbumin using the structural framework obtained from the experimentally solved hen egg-white lysozyme. The hypothesis that drove the study, which has since become a crucial component of TBM, was that since the two proteins shared high sequence homology, they should also be structurally similar. Using this hypothesis, the authors first manually aligned the sequences of both proteins in order to maximize the homology between the two. Following alignment, the authors built a wire skeletal model for hen egg-white lysozyme, whose structure was experimentally determined and then modified it to accommodate the sequence of bovine alpha-lactalbumin, copying the aligned regions and modifying the local structure of the unaligned regions. Although this early attempt utilized a rudimentary approach, it illustrates the four key steps of TBM methods: (1) identification of experimentally solved proteins (templates) related to the protein to be modeled, (2) alignment of the protein of interest and the templates, (3) construction of the initial structural framework by copying the aligned regions, and (4) construction of the unaligned regions and refinement of the structure.

**Figure 2:**
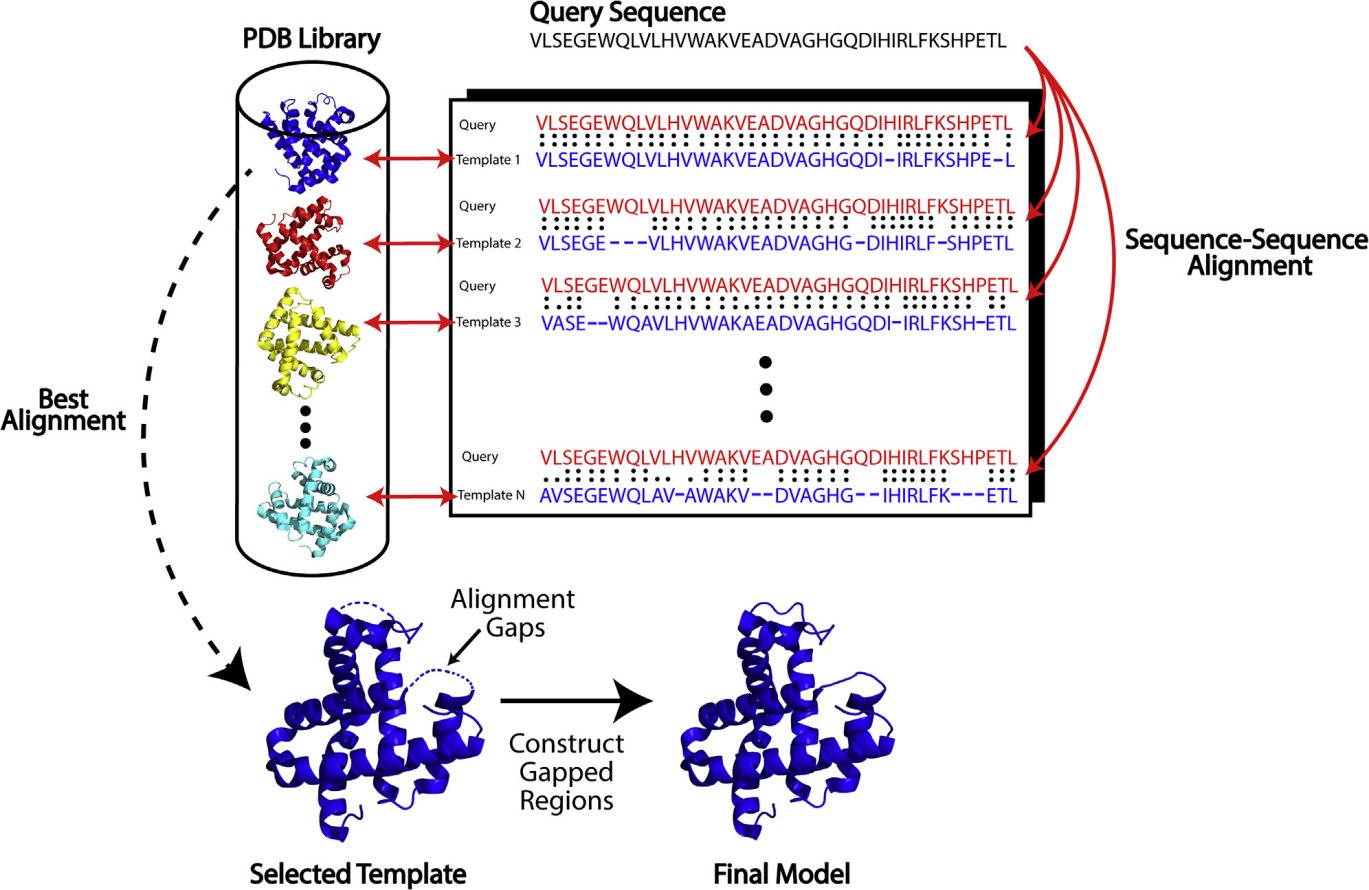
**Typical steps in a homology-based modeling pipeline.** Starting from a query sequence, templates are identified using sequence-based alignment algorithms. Then the structural framework of the best template alignment is copied, and the unaligned regions are constructed to produce the final model.

The case highlighted above for bovine alpha-lactalbumin falls under a special category of TBM called homology modeling or comparative modeling, which typically can be used when the sequence identity between the template and protein of interest is high (*e.g.*, ≥30%). This makes it significantly easier to identify high-quality templates and produce reliable alignments using simple sequence–sequence alignment algorithms. Such algorithms include well-established methods developed in the 1970s and 1980s that utilize dynamic programming, such as the Needleman–Wunsch algorithm (85) for global alignment and the Smith–Waterman algorithm (86) for local alignment. In addition to relatively slow dynamic programming-based methods, rapid sequence–sequence alignments can be obtained using the popular BLAST software (87), which was developed in 1990 and works by first heuristically identifying short matches between the query and template and then attempting to extend these matches to obtain alignments. Once the template and protein of interest have been aligned, the next step is to construct a model by copying and refining the template’s structure. One early approach for constructing homology models that was published in 1993 and has remained popular to the present day is MODELLER (20). MODELLER builds tertiary structure models by optimally satisfying the spatial constraints taken from the template alignments as well as other general structural constraints such as ideal bond lengths, bond angles, and dihedral angles. Figure 2 depicts the main steps involved in a homology modeling approach.

### TBM—threading

The accuracy of homology-based TBM sharply declines on average when the sequence identity between the best available template and the target protein is <30%. Therefore, more advanced alignment approaches beyond simple sequence–sequence-based methods are necessary to identify and obtain accurate template alignments for these cases. In 1991, Bowie *et al.* (18) published their seminal paper that directly addressed this problem by matching 1D sequences to 3D template structures, which has since launched a major research field in the broader domain of TBM known as “threading” or “fold recognition.” The hypothesis that drove the work by Bowie *et al.* was that the 3D structure of a template could be decomposed into a 1D profile of local structural features, which should be more conserved than the amino acid identities themselves and could be used to identify and align proteins with similar structures but more distant sequence homology. Along these lines, the authors categorized each template position into different environmental classes based on the buried/exposed surface area and local secondary structure at a position and then derived a score for finding each amino acid in the different environmental classes. Finally, they integrated these scores into a dynamic programming algorithm to obtain more accurate query-template alignments for distantly homologous proteins.

Another strategy for identifying distantly related proteins was published in 1997 and extended the BLAST methodology to PSI-BLAST (88). PSI-BLAST works by first constructing a multiple sequence alignment (MSA) using sequences detected by a BLAST search. This alignment is then converted into a position-specific score matrix (PSSM), which captures the amino acid tendencies at each position of the MSA and is used in place of the query sequence to iteratively search through a sequence database a prespecified number of times using an algorithm that is similar to that of BLAST. After each step, the profile or PSSM is updated to reflect the sequences detected in the previous round. Thus, the idea behind PSI-BLAST is to iteratively search a database using profiles, which more fully represent the sequence space compatible with a given protein fold, in order to detect more distantly related proteins. Besides PSSMs, sequence profiles may be represented using profile Hidden Markov Models (HMMs). Here, a profile HMM is a probabilistic model that encapsulates the evolutionary changes in an MSA. The advantage of using profile HMMs is that they use position specific gap penalties and substitution probabilities, which more closely represents the true underlying sequence distribution (89). Profile HMMs were introduced in structural bioinformatics in 1994 (90) and have remained one of the most effective methods for identifying templates and constructing MSAs (89, 91, 92).

Most current threading algorithms combine the ideas behind both the approach of Bowie *et al.* and PSI-BLAST by using local structural features, either predicted for the protein of interest or derived from templates, and sequence profiles, represented by PSSMs or HMMs, to identify distantly homologous templates for a given protein sequence (91, 93, 94). In addition, the most recent progress in the field is to integrate contact and distance predictions into dynamic programming-based threading methods to improve the ability of distant-homology template recognition (95, 96), which will be discussed later. Furthermore, meta-threading approaches such as 3D-Jury (97) and, more recently, LOMETS (98, 99) combine the templates output by multiple threading programs into a set of consensus templates. While rigorous theoretical studies to explain the consistent improvement brought about by combining multiple structures were not available until many years later (100), the intuition behind the usage of multiple threading templates is simple. Since there are many more ways for threading to get incorrect alignments than to get a correct alignment, it is much easier to get a consensus correct alignment than multiple consistent but incorrect alignments (101).

### TBM—building tertiary structure models from threading templates

While threading may be used to identify templates for a protein of interest, the output of such programs is an alignment between the query protein and the threading templates, which in and of itself does not provide a 3D model. Therefore, it is necessary to use methods that are capable of converting threading alignments to 3D models in order for the information to be useful. Moreover, the identification of less reliable templates for nonhomology modeling targets makes the construction of 3D models more difficult, necessitating more effective protein structure prediction algorithms that are capable of fixing alignment errors and large alignment gaps. Here, it is worth noting that many homology modeling approaches today also start from templates identified by threading approaches and use more sophisticated model construction techniques than simple homology methods. One successful strategy for modeling distant-homology protein targets is TASSER (32). Developed in the early 2000s, TASSER extracts contiguous fragments from the threading aligned regions of multiple threading templates, which are then reassembled during its structure assembly simulations. For computational efficiency, the unaligned regions are assembled using a lattice-based FM approach. In addition to constraints from template alignments, TASSER also incorporates several knowledge-based energy terms important for protein folding (*e.g.*, hydrogen bonding, secondary structure formation, side-chain contact formation, etc.) to guide its parallel hyperbolic Monte Carlo simulations (102). Following the simulations, low-energy decoys are clustered based on their structural similarity, and the largest cluster centroid is selected for additional full-atom refinement. The key reason for the success of TASSER is its effective combination of multiple templates (20, 21, 22, 23, 24, 25, 26, 27, 28, 29, 30, 31, 32, 33, 34, 35, 36, 37, 38, 39, 40, 41, 42, 43, 44, 45, 46, 47, 48, 49, 50) and its optimized simulation strategy that combines efficient conformational movements with an effective knowledge and template-based energy function.

**Figure 3:**
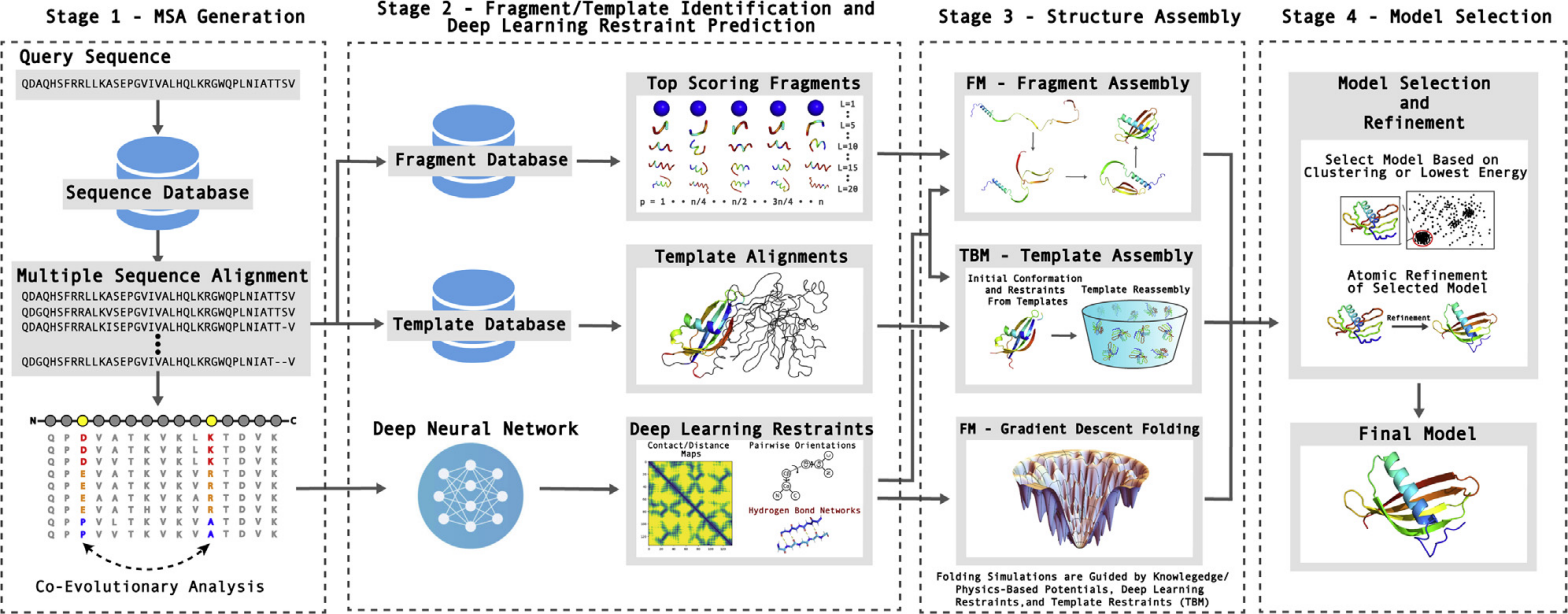
**Typical steps in template/fragment assembly and gradient descent-based protein structure prediction pipelines.** Starting from a query sequence, a multiple sequence alignment (MSA) is constructed by identifying homologous sequences from a sequence database. Then using profiles or predicted structural features derived from the MSA, either global template structures (for TBM) or local fragments (for FM) are identified from databases of solved protein structures. Additionally, coevolutionary analysis of the MSA is fed into deep neural networks to predict pairwise restraints such as distance maps, interresidue orientations, and hydrogen bond networks. The structure assembly stage may either assembly the local fragments, global template structure, or directly minimize the structure using rapid gradient descent methods. From here, the final model may be selected by clustering the conformations generated during the structure assembly stage or by identifying the lowest energy structure, which is further refined using atomic-level refinement simulations to produce a final model.

More recently developed TBM approaches such as I-TASSER (22, 24, 103), RosettaCM (104), and Phyre2 (105) also combine constraints from multiple templates. For example, I-TASSER, which is an extension of TASSER, uses multiple templates identified by LOMETS; compared with TASSER, the main difference of I-TASSER is that, following clustering of the low-energy decoys and selection of the cluster centroid, the centroid is searched through the PDB library to identify additional templates. Constraints from these templates, the cluster model, and the threading templates are combined with the inherent knowledge-based potential to guide a second round of structure assembly simulations. Following this, the lowest energy structure is selected and subjected to full-atom refinement. Since its introduction in CASP7, I-TASSER has been consistently ranked as the top automated protein structure prediction server, where it was one of the first methods to regularly demonstrate the ability to draw template structures closer to the native structure. Here, CASP is the community-wide blind modeling experiment to determine the state of the art in protein structure prediction, which has taken place every other year since 1994 (106). The motivation for establishing the experiment was to provide an objective means of evaluating the state of the field and measuring the performance of various proteins structure prediction approaches. Before the introduction of I-TASSER in CASP7, the CASP assessors concluded, “We are forced to draw the disappointing conclusion that, similarly to what [was] observed in previous editions of the experiment, no model resulted to be closer to the target structure than the template to any significant extent” (107) and “Sad notes are once again those regarding the poor performance in predicting features not directly inheritable from the parent and in obtaining a model that is closer to the native structure than the template used to build it” (107). Thus, the ability to draw template structures closer to the native represents a significant achievement and a solution to one of the classical problems in TBM. Figure 3 depicts the main steps involved in a generic threading-based protein structure prediction algorithm.

One factor that may or may not be considered by threading approaches is the resolution of the templates themselves, which is a measure of how closely the experimental structures match the native structures. For example, programs such as LOMETS search a nonredundant structure library that consists of templates of varying resolutions obtained by methods including X-ray crystallography, electron microscopy, and NMR spectroscopy. Since the vast majority of the structures in the PDB were determined by X-ray crystallography (~88%), followed by NMR (~7.5%), and then electron microscopy (~4.3%), the most frequently used templates are those determined by X-ray crystallography, which also typically have better resolutions than NMR and electron microscopy structures. The templates are then ranked according to the query-template alignment scores obtained by the component threading programs, without regard to the resolution of the selected templates. However, it must be noted that the experimental resolution of structures in the PDB is in general high-quality compared with many predicted models, especially those that lack close homologous templates, where only approximately 1.5% of PDB structures have resolutions worse than 4.6 Å and around 42% have resolutions better than 2 Å. Nevertheless, for homology modeling studies, it is important to select higher-resolution templates when multiple structures are solved using different techniques by different laboratories for the same protein, as the final structures closely match the initial templates.

As the success of TBM relies on the availability of PDB templates, the average quality of the TBM models varies depending on the type of protein being modeled. For example, given the difficulty in determining the crystal structures of certain classes of protein, such as GPCRs (or membrane proteins more generally) and proteins with disordered regions, some structures may be more difficult to model by TBM as fewer templates are available (108, 109). To help partially overcome this, specialized methods have been designed to predict structures for these classes of proteins (110, 111).

### FM—molecular dynamics

FM protein structure prediction methods generate models without using global template structures. These approaches typically try to find the lowest energy conformation for a protein structure using an energy function that accounts for forces that are fundamental to protein folding, as, based on Anfisen’s thermodynamic hypothesis (1), the native structure of a protein should be its lowest free energy conformation. The earliest attempts at FM were implemented to refine the atomic structures produced by X-ray diffraction experiments in order to improve their physical characteristics. For example, the method by Levitt *et al.* (112) published in 1969 combined an energy function that accounted for typical bond length, bond angle, and dihedral angle values as well as the van der Waals interactions and restraints taken from experimental structures with a steepest descent-based minimization procedure to refine the X-ray structure of lysozyme and myoglobin. A similar energy function was used in 1977 by Karplus’ group to study the dynamics of the bovine pancreatic trypsin inhibitor (17). The authors used a molecular dynamics approach to study the motion of the protein, where the goal of molecular dynamics is to solve Newton’s second law for all atoms in a system over a given time period to determine their motion. Since then, various molecular dynamics force fields and packages have been developed including AMBER (113, 114, 115), CHARMM (116, 117, 118), OPLS (119, 120), and GROMOS96 (121). Despite their different parameterizations, all of these potentials bear a resemblance to the original potential developed by Levitt *et al.* in 1969 in terms of their functional forms. Although molecular dynamics is useful for refining the atomic structures of proteins, it is very difficult to apply it to predict a protein structure starting from sequence. This is perhaps best illustrated by the fact that the first successful molecular dynamics-based protein structure prediction was generated in 1998 by Duan and Kollman for the very small villin headpiece subdomain (36 amino acids), which took 2 months CPU time to simulate using a massively parallel supercomputer and achieved an accuracy of 4.5 Å (115). Since then, technology has progressed through the use of more advanced computer architectures (122, 123) and force fields (124, 125, 126, 127, 128, 129, 130), but molecular dynamics-based structure prediction still remains impractical for proteins of typical length. Nevertheless, molecular dynamics has remained a popular tool to study protein motion (128, 131) and for full-atom refinement of protein structures (132, 133, 134).

### FM—fragment assembly

Besides molecular dynamics-based methods, many current FM approaches use fragment assembly, an idea pioneered by Bowie and Eisenberg in 1994 (135). The implementation by Bowie and Eisenberg generated a mixture of fragments with fixed (nine residues) and variable lengths (15–25 residues) from a database of known 3D structures. Fragments were chosen based on their compatibility to the sequence of the protein they wanted to model, where compatibility was assessed using the profile-based threading method developed earlier by Bowie *et al.* These fragments were then used to assemble full-length structural models for small alpha-helical proteins. The authors found that this fragment assembly procedure reduced the conformational search space, while ensuring that the local structures of the assembled fragments were well formed. Following the idea of Bowie and Eisenberg, Baker’s group developed the Rosetta modeling software in 1997 (21), which has remained one of the most widely used FM methods to this day. In Rosetta (136), three and nine residues fragments are scored based on the profile-profile and secondary structure similarity between the query sequence and fragments over a selected window size. The main conformational move is fragment insertion, where the backbone torsion angles of the predicted conformation are swapped for those of one of the high scoring fragments during a simulated annealing Monte Carlo simulation. Structures are represented using a course-grained model that explicitly models all backbone atoms and the side-chain centers of mass. A centroid energy function is employed to guide the simulated annealing Monte Carlo simulation, which includes terms that account for important factors in protein folding such as helix-strand packing, strand pairing, solvation, van der Waals interactions, radius of gyration, strand arrangement into sheets, and residue pair interactions. Conformations generated during the simulation with favorable local interactions and protein-like global properties are clustered based on their structural similarity, and the final structure is typically derived from the largest cluster center. Apart from the Rosetta, additional FM predictors, such as QUARK (23) and FragFold (137), were developed by other groups based on a similar idea of fragment assembly using variants of Monte Carlo simulations, but with different approaches for fragment generation and energy function design. For example, QUARK includes a distance-based profile energy term, which estimates and constrains the distance between two residues based on the interresidue distances of fragments taken from the same PDB structures. Moreover, QUARK includes a set of 11 different conformational movements in addition to the fragment replacement movement, making the conformational sampling procedure more efficient. Since Rosetta’s introduction in CASP3 and QUARK’s introduction in CASP9, they have been consistently ranked among the top FM approaches. Figure 3 depicts the main steps involved in a generic fragment assembly-based FM approach.

### FM—rapid gradient descent-based folding methods

While fragment assembly represents one successful approach used by FM methods, the drawback is that the simulations may take several hours to days depending on the length of the protein. Therefore, it is desirable to develop methods that are capable of generating structures rapidly. This can be achieved using gradient descent-based folding methods. One limitation of such approaches, however, is that they may be prone to becoming trapped in local minima, as opposed to finding the conformation that lies at the global minimum of the energy distribution. This is particularly true when the energy landscape is complex, which is the case in protein folding. Recently, this problem has been addressed through the accurate prediction of pairwise spatial restraints, such as interresidue distances, using deep learning, which can smooth the energy landscape and allow gradient-based methods to accurately fold protein structures (29). In CASP13, the first iteration of AlphaFold was able to achieve state-of-the-art performance using a gradient descent-based folding approach (28). Interestingly, they found that the performance of their gradient descent-based pipeline was faster than and achieved similar performance as their fragment assembly approach. Furthermore, trRosetta, the latest iteration of the Rosetta modeling software, uses an L-BFGS gradient descent approach to rapidly fold protein structures and demonstrated that high-accuracy predictions may be achieved even with rapid simulations due to accurate deep learning-based restraints (29). Figure 3 depicts the main steps involved in a generic gradient descent-based FM approach. Here, a critical characteristic for the gradient descent search to work properly is the high number of deep learning-based distance and orientation restraints (typically >20–50*L*, where *L* is the target length) that can simplify and smooth the energy landscape so that the global minimum can be readily recovered even with local conformational searching. For the cases with no or sparse spatial restraints, more advanced structural assembly and conformational searching approaches have been proven to be necessary due to the roughness of the physics- and knowledge-based energy landscapes (21, 23, 138).

## Pairwise spatial restraint prediction

**Figure 4:**
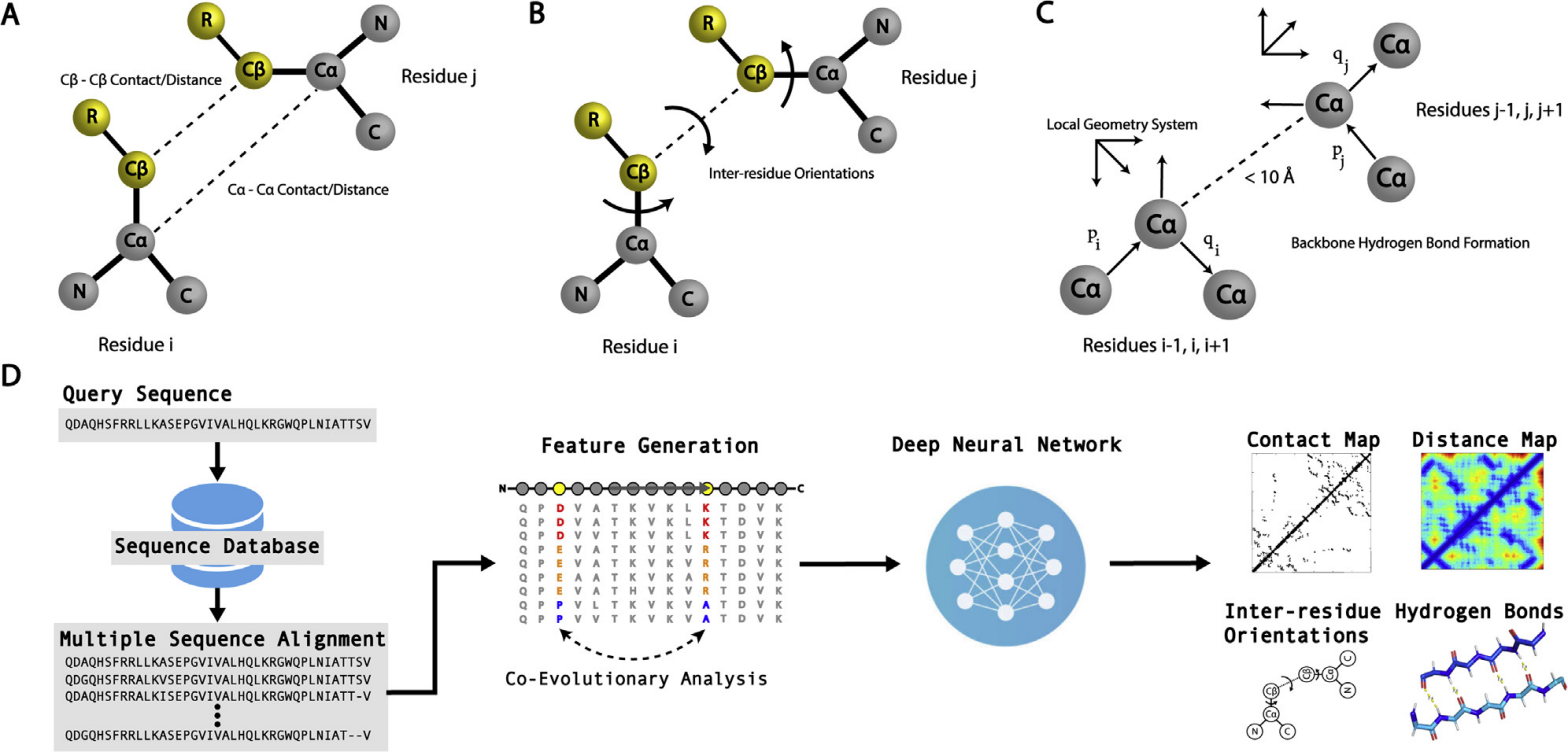
**Interresidue spatial restraints that are often used to assist protein 3D structure assembly simulations.** The protein backbone atoms include the N, Cα, and C atoms, while the side chains include the Cβ atoms, with the exception of glycine, as well as the R groups, which distinguish the different amino acid residues. *A*, C*α*/C*β* contacts and distances; *B*, interresidue torsion angles; *C*, hydrogen bond networks. Here, the backbone hydrogen bonds are represented using a Cα-based model, where three consecutive Cα atoms form a local coordinate system, from which various vectors and their orientations represent regular hydrogen bonding patterns observed in native proteins. *D*, typical pipeline for spatial restraint prediction. Starting from the amino acid sequence of a target protein, homologous protein sequences are collected from sequence databases and compiled to form a multiple sequence alignment (MSA). For the MSA, coevolutionary relationships are deduced and fed into a deep neural network, which may output the predicted contact/distance maps, interresidue orientations, and hydrogen bond networks.

The use of deep learning techniques to predict pairwise spatial restraints has become a major area of research in the field. This is because the tertiary structures of proteins are formed and stabilized by interactions between the atoms that make up each residue, and prediction of these interactions provides extremely useful information that can guide protein folding approaches. Perhaps the most commonly predicted interactions are those between C_β_ atoms from different residues. In general, two residues are considered to form a contact if the distance between their C_β_ atoms (C_α_ for glycine) is <8 Å, where an illustration of contacts/distances in protein structure prediction is depicted in Figure 4*A*. Here, a contact map for a protein with length *L* is a symmetric, binary *L* × *L* matrix, where each element of the matrix is a binary value that indicates if the residues form a contact or not. The concept behind distance maps is similar, but they provide more detailed information on the interactions. Instead of simply predicting if two residues are in contact or not, distance map prediction attempts to directly predict the distance between two atoms from different residues, typically the C_β_ atoms or C_α_ atoms for glycine. In practice, most distance map predictors do not predict the exact distance between residues, but the probability that the distance falls within a certain range. Although inclusion of contact and distance maps predicted using deep learning has recently transformed the field of protein structure prediction, the prediction of residue–residue contacts/distances is not a new idea.

Beginning in the 1990s, attempts were made to predict the residue–residue contacts for a protein based on correlated mutations in MSAs (139, 140, 141). The hypothesis behind the approach was that if mutations that occur at two positions are correlated, they are more likely to form a contact in 3D space; this is because there is evolutionary pressure to conserve the structures of proteins. Therefore, a mutation at one position along the sequence that may result in structural instability could be rescued by a corresponding mutation in a residue that is in contact with the mutated residue. As a result, it would be expected that residue pairs that are in contact would exhibit correlated mutation patterns, also known as coevolution. In practice, however, the accuracy of early covariation-based approaches was limited by the inability to distinguish between direct and indirect interactions. An indirect interaction may occur when position *A* forms a direct contact with position *B* and *B* forms a direct contact with position *C*; even if *A* does not directly contact *C*, coevolution may still be observed between positions *A* and *C*. This is because a mutation at position *A* may cause a mutation at position *B*, which in turn could result in a compensatory mutation at position *C*, thus appearing as if positions *A* and *C* coevolve. Further restrictions were imposed by the limited size of the sequence databases used to construct MSAs and the lack of sophisticated MSA construction methods.

### Improving contact prediction through the use of global statistical models

The inability to distinguish between direct and indirect interactions remained a significant challenge until contact prediction algorithms began using global prediction approaches. The first contact prediction methods considered one residue pair at a time using techniques such as mutual information (141), thus ignoring the interactions with other residue pairs and the global context in which the interactions took place. The introduction of global statistical models determined through the use of direct coupling analysis (DCA) was much more successfully able to distinguish between direct and indirect interactions (142, 143). The improved performance of DCA over mutual information and other related methods is due to the fact that DCA simultaneously considers the full set of pairwise interactions, instead of considering residues one at a time. A widely used DCA method is to fit a Markov random field (MRF), or more specifically a Potts model, to an MSA using message passing (142), Gaussian approximation (144), mean-field approximation (143), or pseudo-likelihood maximization (145, 146, 147). Here, an MRF model represents each column of an MSA as a node, where the determined edge weights between each node can be used to infer contacts between each position. Other popular methods include estimation of the inverse covariance matrix, also known as the precision matrix, from an MSA using L1 regularization, as introduced by PSICOV (148), or L2 regularization, as introduced by ResPRE (149). Network deconvolution has also been used to determine contacts from coevolutionary data (150).

### Contact map prediction using shallow machine learning approaches

While the use of DCA represents a promising avenue to improve contact prediction accuracy, another approach is to leverage machine learning to predict the interresidue contacts and distances. In fact, the use of machine learning in contact prediction dates back as far as simple covariation-based techniques. Early machine learning methods utilized shallow, fully connected neural networks, whose inputs included features such as correlated mutation data, secondary structure, and sequence conservation information (151, 152). Here, the distinction between shallow and deep neural networks is primarily based on the number of hidden layers in the network, where shallow networks have few hidden layers. These early machine-learning-based predictors achieved comparable or slightly better accuracies than the contemporaneous methods based solely on analysis of correlated mutations. Following the first iteration of machine-learning-based contact predictors, more complex neural network architectures were developed (153, 154, 155, 156). Furthermore, contact prediction methods based on other machine learning techniques such as support vector machines (SVMs), including SVMSEQ (157) and SVMcon (158), or random forest models, including PconsC (159), achieved success by extracting a large number of features for a target protein sequence and then applying SVMs/random forests to solve the classification problem. Success was also reported by meta-methods such as MetaPSICOV (160) and NeBcon (161), which combined the output of multiple DCA methods using shallow neural networks and could outperform the best individual component programs.

### Contact map prediction using deep neural networks

In the early 2010s, predictors began to incorporate deep learning architectures into their prediction methods. The first of these included CMAPpro (162), which used a 2D recursive neural network, and DNCON (163), which used a deep belief network. Such networks achieved accuracies similar to or better than other state-of-the-art predictors at the time, such as SVMcon and PSICOV, but the accuracies were still relatively low; in fact, early deep learning approaches could not outperform MetaPSICOV, a shallow neural network approach trained on multiple DCA predictors. Part of the reason for the suboptimal performance of these deep learning networks was that contacts for pairs of residues were predicted using features extracted only from a small window of residues around the target residue pair. This sliding window approach ignores the global context of the residue pairs, therefore not realizing the true potential of deep learning.

A breakthrough came in 2017 when Xu’s group proposed RaptorX-Contact (26), which reformulated the contact prediction problem through the introduction of deep residual convolutional neural networks (ResNets (164)), where a representative pipeline for deep learning-based spatial restraint prediction is shown in Figure 4*D*. Here, a residual neural network is a convolutional neural network that adds an identity map of the input to the output of the convolutional layer, allowing gradients to flow smoothly from deeper to shallower layers and enabling the training of deep networks with many layers. Under this framework, the contact map prediction problem is considered an image segmentation task, *i.e.*, a pixel-level labeling problem, where the whole contact map is an image in which each residue pair corresponds to a pixel. Image segmentation is a task for which ResNets, originally developed for computer vision, have demonstrated excellent performance. While the features used by RaptorX-Contact, such as coevolutionary information obtained through DCA, predicted secondary structures, and PSSMs, are quite similar to other predictors, the introduction of deep ResNets with approximately 60 hidden layers enabled RaptorX-Contact to dramatically outperform other methods. The demonstrated power of ResNets has inspired the vast majority of top ranked methods (165, 166, 167) developed since CASP12 to incorporate them into their architectures. One particularly successful method in CASP13, TripletRes (168, 169), used a similar ResNet basic block for its deep learning architecture but with a triplet of coevolutionary matrices. Instead of using the postprocessed *L* × *L* evolutionary coupling information utilized by other predictors, TripletRes directly used the 21 × 21 × *L* × *L* raw coupling parameters as an input feature to its network, where 21 is the number of amino acid types (plus one type for gaps). The usefulness of deep learning-based contact map prediction was clearly demonstrated by C-I-TASSER and C-QUARK in CASP13, which were ranked as the first and second best automated servers in CASP13, respectively (27). C-I-TASSER and C-QUARK were extensions of the classic I-TASSER and QUARK frameworks, which included contact maps from TripletRes, ResPRE, and numerous deep learning-based predictors into their simulations. These deep learning restraints were found to greatly improve the modeling accuracy, especially for targets without readily identifiable template structures (27).

### Distance map prediction using deep learning

A natural extension of contact map prediction is distance map prediction. The difference between the two is that contact map prediction involves binary classification, while distance map prediction typically involves multiclass classification. In other words, instead of predicting if two residues form a contact or not, distance map prediction typically predicts the probability that the distance between residues falls into one of many different bins (even though attempts have been made to directly predict the real-value distances (170)). We note that the idea of distance prediction is not new; QUARK (171), for example, includes distance predictions derived from fragments detected from templates. Yet, the implementation of distance prediction in a deep learning framework is a recent advancement and makes the prediction much more robust and successful even in the absence of analogous structural templates. Distance map prediction jumped to the forefront of the field during the CASP13 experiment in 2018, when three predictors (RaptorX-Contact (43), DMPfold (172), and AlphaFold (173)), extended the use of deep ResNets for contact prediction to distance prediction. Of these predictors, AlphaFold achieved the best performance in tertiary structure modeling, as it was ranked as the top human group in CASP13. Starting from the coevolutionary coupling information obtained from an MSA, AlphaFold utilized a very deep residual neural network composed of 220 residual blocks to predict the distance map for a target sequence, which was then used to assemble protein models.

In CASP14, distance map prediction was prevalent among the top predictors and has replaced contact map prediction to a large extent as the information encoded in distance maps is much richer than that in binary contact maps. Top distance map prediction approaches that participated in the contact prediction section of CASP14 include DeepPotential (174) and tFold (175). Here, it is important to note that contact maps can be obtained from distance maps by collapsing the predicted distance maps into binary matrices, thus allowing them to be assessed in contact map prediction. DeepPotential used a deep ResNet composed of 50 2D residual blocks to simultaneously predict pairwise distance maps, interresidue orientations, and hydrogen bond networks (Fig. 4). Interestingly it was found that training on multiple features such as interresidue distances and orientations, which are discussed in the next section, improved the distance map prediction performance. Similarly, tFold also predicted pairwise distances and orientations using a deep ResNet. However, tFold’s network was composed of more than ten times the number of layers of DeepPotential, with 600 residual blocks, and utilized a 2D attention mechanism. Of note, the developers found that the utilization of 600 layers was able to improve the performance slightly, suggesting that there is a steep diminishing return on investment when adding additional layers. Although it did not participate in the contact prediction section of CASP14, another successful distance map prediction approach was trRosetta, which was developed before the CASP experiment and uses a deep ResNet to predict both pairwise distances and orientations (29).

### Interresidue orientation and hydrogen bond network prediction using deep learning

A further extension of distance prediction is interresidue torsion angle orientation prediction. It has been known for years that knowledge-based energy functions that are dependent only on residue-residue distances are often not as accurate as those that use both residue–residue distances and orientations for protein structure prediction (176, 177, 178). The importance of orientation-dependent energy functions is twofold: biologically, certain types of residue–residue interactions require not only distance proximity but also specific orientations between the residue pairs, *e.g.*, beta strand pairing; mathematically, it is impossible to uniquely determine the geometry of a structure without torsion angle information, as distance information alone cannot differentiate a pair of mirrored structures. Given the importance of interresidue orientations, a number of structure prediction approaches have incorporated them into their pipelines. For example, NEMO used deep learning to simultaneously predict pairwise distance maps, interresidue orientations, and dihedral angles for a given sequence (179). Interestingly, they incorporated these into an end-to-end learning approach, which directly generated structures using machine learning as opposed to incorporating these restraints into gradient descent or Monte Carlo–based folding simulations.

More recently, trRosetta (29) has popularized orientation prediction by using a deep residual neural network to predict both pairwise residue distances and interresidue orientations from coevolutionary information (Fig. 4*B*). In CASP14, many of the top groups, including Rosetta (180), D-I-TASSER (181), and D-QUARK (182), utilized orientation and distance restraints predicted by deep residual neural networks. In addition, the top CASP14 server group, D-I-TASSER, also used DeepPotential’s residual neural network to predict hydrogen bond networks (Fig. 4*C*) and incorporated the hydrogen-bonding restraints into its structural assembly simulations. The deep learning-based hydrogen bond network prediction was found to significantly improve the modeling accuracy on CASP14 targets, especially for those target that lacked homologous templates (181).

### Incorporating metagenomic sequence data into prediction approaches

Another limitation of the early-stage contact prediction approaches was the small number of homologous sequences that could be used to construct MSAs for a distant-homology target. DCA methods in particular, and deep learning approaches to a lesser extent, rely on collecting a sufficient number of homologous sequences in an MSA, as the more homologous sequences there are, the more reliable the derived coevolutionary information is. Fortunately, the implementation of DCA and deep learning contact/distance prediction has coincided with the expansion of sequence databases, in particular metagenomics sequence databases. Metagenomics is the application of next-generation shotgun sequencing techniques to sequence the DNA collected from environmental samples. These DNA sequences can be translated to protein sequences automatically, thereby producing large databases with billions of protein sequences. The utility of metagenomics sequences in contact-assisted structure prediction was first demonstrated for GREMLIN/Rosetta (25) by significantly enhancing the number of effective sequences in an MSA, thus producing “deep” MSAs with diverse sequences for DCA. Later MSA construction methods (183, 184) confirmed the usefulness of metagenome-derived MSAs for improving contact prediction (168, 183, 184), threading results for distantly homologous targets (99, 184), and the ability to model proteins that belong to families with unknown structures (25, 185).

As a side effect of the rapid accumulation of metagenome data for protein structure prediction, comprehensive sequence database search, and MSA collection has become increasingly infeasible due to both computer speed and memory limitations. Peng *et al.* (186) recently utilized 2.4 TB of the microbiome sequencing data, representing 4.25 billion microbiome sequences and covering four major biomes (gut, lake, soil, and fermentor), to investigate the inherent link between the microbiome niches and their homologous protein families. Their study showed that an MSA searched from an individual biome that is predicted to be most closely linked with the target protein family could result in more accurate contact map prediction and 3D models with higher TM scores, compared with those collected from the combined metagenome samples. This is in spite of the fact that the former used a much smaller metagenome sample with significantly less CPU memory costs than the latter. The rationale lies in the assumption that accurate evolutionary information should be derived from MSAs collected from evolutionarily close genome samples, while the involvement of irrelevant genome samples, although increasing the volume of homologous sequences, can introduce “noise” into the MSA collection and the subsequent contact and distance map prediction procedures. This result provides a promising avenue to curtail the extremely high-volume sequence database search requirement for high-quality structure prediction by using a targeted approach built on the linkage between microbiomes and a target protein’s homologous families.

### Identifying threading templates by contact/distance map-guided threading approaches

Apart from their direct use as restraints to guide protein assembly simulations, contact and distance maps can be used by threading approaches to identify structural templates for a query sequence. In fact, contact and distance map-guided threading approaches represent the state of the art in fold recognition, achieving superior accuracy to traditional profile or local structural feature-based threading approaches (95, 96, 187). This is in part because correct contact and distance maps provide a clear description of a protein’s global fold and may be predicted for a query sequence with high accuracy using deep learning or obtained from the native template structures. However, aligning two contact maps is a nontrivial problem, and various methods have been developed to address this critical issue. Among them, EigenTHREADER (96) uses eigen decomposition of contact maps to obtain the top eigenvectors, then the template and query contact maps may be aligned by aligning their principal eigenvectors. CEthreader uses a similar eigen decomposition strategy but goes beyond pure contact map-based threading approaches, incorporating information from both local structural feature prediction and sequence-based profiles (95). Furthermore, map_align (25) proposed an iterative double dynamic programming algorithm to align contact maps, while DeepThreader (187) uses predicted distance maps and the ADMM algorithm to obtain alignments. More recently, DisCovER (188) incorporated deep learning-based distance and orientation prediction into their threading approach, along with a topological network that includes information from neighboring residues, ultimately obtaining alignments using an iterative double dynamic programming framework. One drawback to contact and distance map-guided threading approaches is that they tend to be more computationally demanding, mainly because the interresidue contact/distance maps involve two-body information that cannot be directly integrated in the dynamic programming or hidden Markov models that require single-body potentials. A typical strategy used to overcome this is to first identify a certain number of top templates using rapid profile-based threading methods. Then the identified templates may be realigned using the contact and distance map-based approaches, thus reducing the number of costly alignments that must be performed (95).

### Unsupervised contact map prediction using transformers

Although MRF models or Potts models have been shown to be useful for predicting pairwise spatial restraints, they are not without their drawbacks. One very critical drawback is their dependence on identifying a relatively large number of homologous sequences in order to ascertain coevolutionary relationships in an MSA. Although deep residual neural networks have partially alleviated this issue, the problem remains as there still exists a considerable correlation between the number of effective sequences (Neff) in an MSA and the prediction accuracy. Additionally, MRF models are essentially a human-engineered feature used by most deep learning approaches, which somewhat violates a key aspect of deep learning in that the networks themselves should extract useful features.

Recently, exciting progress has been witnessed in unsupervised contact prediction using self-attention-based deep learning architectures called transformers. Transformers are a novel machine learning architecture that was introduced in 2017 and have significantly impacted the field of natural language processing, outperforming recurrent and convolutional networks (189). Briefly, transformers pass inputs through a series of self-attention and feedforward connections, which allow the network to attend to relevant information from the input and build up complex representations that incorporate long-range dependencies. Rives *et al.* (190) first applied transformers to contact prediction by training a transformer model to recover masked amino acid types for 86 billion residues from 250 million protein sequences. Although the model was not specifically trained to predict contact maps, they can be deduced from the information encoded in the final hidden representation learned by the transformer model. Thus, using this approach, contact maps may be predicted in an unsupervised manner, allowing training on protein for which no structural information is available.

**Figure 5:**
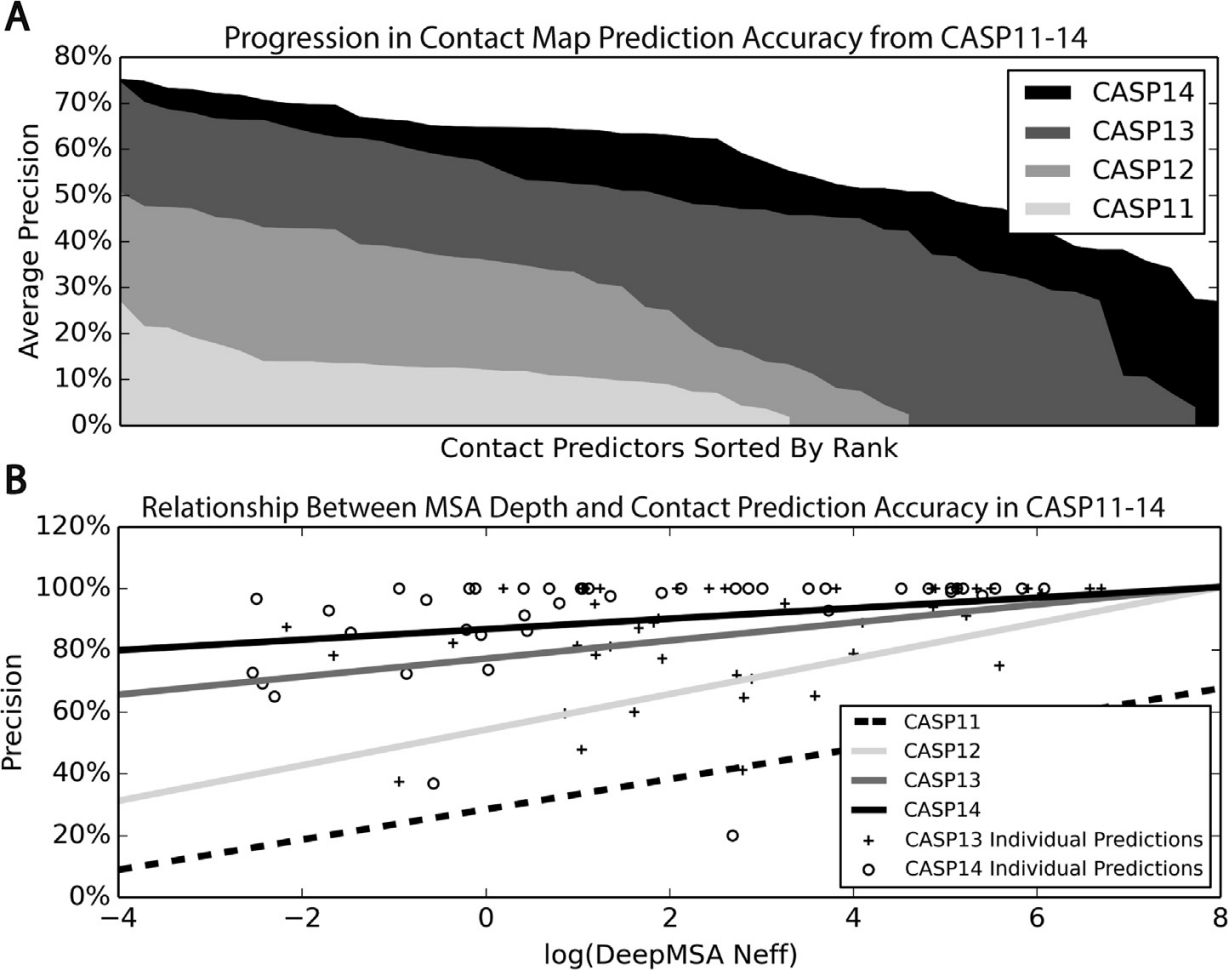
**Summary of contact map prediction results in CASP11 to 14.***A*, contact prediction results for different groups on all FM and FM/TBM targets. Groups are sorted in descending order of the average precision of their top *L*/5 long-range contacts, where *L* is the protein length and long-range contacts occur between positions that are separated by at least 24 residues. *B*, relationship between contact prediction precision and the MSA Neff value obtained by the DeepMSA program (184), where lines are the best fit on the individual targets by linear regression.

The single sequence model was recently extended to MSAs, outperforming current state-of-the-art methods such as trRosetta on contact prediction (191). Of particular interest is the improvement in performance for targets with MSAs composed of few effective sequences, which have traditionally been more difficult prediction targets as determining coevolutionary information using MRF models requires many sequences in an MSA. This is similar to what was observed in CASP14 by AlphaFold2 (77), which also used self-attention to arbitrarily attend to sequences from an MSA and pick up relevant information in the Trunk section of their network. The goal of the novel transformer architecture introduced by AlphaFold2 is to treat the protein structure prediction problem as a graph inference problem, where residues that are close together in 3D space define the edges of the graph using both a pairwise and MSA representation. Here, the pairwise representation is used to represent the spatial proximity of each pairwise interaction between residues and the MSA representation encodes the evolutionary information from the detected sequence homologs. The AlphaFold2 Trunk network consists of multiple blocks, where at the beginning of each transformer block, the MSA representation is processed using multiple self-attention layers, and the attention mechanism is biased by the pairwise representation to ensure proper communication and consistency between the two. Then the processed MSA representation is in turn used to update the pairwise representation. Since the pairwise interactions or edges must satisfy the triangle inequality in accordance with the properties of protein structures, the pairwise representation is updated using a triangle self-attention and updating scheme that considers a triangle of edges formed by three residues. The final model quality produced by AlphaFold2 exhibited almost no correlation to the number of effective sequences in the MSAs by DeepMSA (184) (Fig. 5*A*), demonstrating that the problem of low prediction accuracy for targets with few effective sequences may be partially addressed through self-attention-based neural networks, at least based on the MSAs collected from a third-party program.

## End-to-end structure prediction using deep learning

### First attempts at end-to-end folding

While many successful methods to date have focused on predicting pairwise structural features and incorporating them into structure assembly simulations, the ideal approach to solving the structure prediction problem would be to directly learn the 3D structures of proteins starting from their amino acid sequences, the so-called “end-to-end” learning approach. This would remove the need for advanced folding simulations and instead allow deep neural networks to directly produce 3D structures. One of the first attempts at end-to-end deep learning-based structure prediction used recurrent geometric networks to build protein models by predicting the backbone torsion angles for each residue (192). Here, it is important to note that protein structures can be described in terms of the Cartesian coordinates of all of the atoms that make up each amino acid residue or in torsion angle space, assuming ideal bond lengths and bond angles. Representing a protein conformation by its torsion angles allows for the prediction and optimization of significantly fewer parameters. In addition, Cartesian coordinates are more difficult to predict using machine learning as rotating or translating a structure will result in significantly different coordinates for the same protein structure. Thus, a representation that is not dependent on arbitrary translation or rotation is needed to achieve self-consistency, which is why the method used the torsion angle representation of protein structure. A drawback of the torsion angle representation is, however, that any small error at a local residue may result in a big RMSD error for the global structure. The deep neural network was made up of stacked long short-term memory (LSTM) units that received position specific amino acid and PSSM information along with information from other upstream and downstream LSTM units. The output of the network was the predicted backbone torsion angles for each residue. From these predicted torsion angles, the backbone structure can be built directly one residue at a time from the N to C-terminal by converting from torsion angle space to Cartesian space using simple geometric functions.

While this may be one of the first claims of end-to-end learning, the idea is similar to predicting the backbone torsion angles for a given query sequence, which is a long-standing idea in the field (193). One of the key differences, apart from the neural network architecture, is that the loss function for training took into account the deviation between the predicted and native structures as opposed to just the error in the torsion angle prediction. Nevertheless, the method performed poorly in CASP13, suggesting that direct prediction of torsion angles alone may not be a robust method for constructing tertiary structure models. This is in part because torsion angles are essentially local features and may not accurately capture long-range information that is critical in structure modeling and small errors in the predicted torsion angles can result in large structural deviations downstream due to lever-arm effects. In fact, AlphaFold used a similar end-to-end network to generate protein structures based on torsion angle prediction in CASP13 and found that indeed the long-range interactions were poorly formed by such networks (173). Ultimately, they used the network to produce short structural fragments, which were then assembled using a distance map-guided fragment assembly approach.

Another method for end-to-end folding that was developed at around the same time as the recurrent geometric network approach is NEMO (179). NEMO uses a combination of 1D, 2D, and graph convolutions to predict interresidue distances, orientations, and dihedral angles and utilizes Langevin dynamics to generate models based on these predicted features. Thus, the approach represented the protein conformation by a combination of the backbone dihedral angles as well as the interresidue distances and orientations. Here it is important to note that similar to the torsion angle representation, a protein structure can be described in a manner that is independent of translation or rotation in 3D space by the full pairwise distance maps, with the exception of mirror image structures. Despite the unique approach, the method was outperformed by more traditional protein folding approaches that used deep learning-based restraints. However, the realization of end-to-end training for protein structure prediction was achieved in CASP14 by the second iteration of AlphaFold, AlphaFold2, which attained remarkable modeling accuracy and has largely solved the single-domain protein structure prediction problem (83).

### End-to-end folding in CASP14 by AlphaFold2

The breakthrough achieved by AlphaFold2 can be in part attributed to their unique end-to-end learning approach, which replaced traditional folding simulations with 3D equivariant transformers (77). The AlphaFold2 structure modeling network consists of two main parts: the Trunk section, which is responsible for processing the input data including the query sequence, templates, and MSA, and the Structure (or Head) Module, which is responsible for directly mapping 3D structures from the training elements (77). The Trunk section of the network is briefly explained in the Unsupervised Contact Map Prediction using Transformers section. More specifically, the main building blocks of the Trunk section are self-attention transformers, which process the MSA and pairwise representations using self-attention networks, where the MSA representation is initialized from the raw MSAs detected from the sequence database searches and the pairwise representation is initialized from the target sequence and pairwise template features derived from the top templates detected by HHsearch. The pairwise representation output from multiple transformer blocks is then fed into the Structure Module along with the row in the MSA representation that corresponds to the original target sequence, which is referred to as the single representation. The Structure Module represents 3D structures using a gas of 3D rigid body frames. Here, a rigid body frame describes the rotation and translation of each residue, where the rotation of the backbone atoms is accounted for by the three backbone torsion angles (φ, ψ, and ω) and the rotation of the side-chain atoms is specified with the side-chain torsion angles ($\chi_{1-4}$). In addition to the torsion angles, the network produces predictions for the translation vectors of each frame. For the backbone frames, which consist of the N-Cα-C atoms, the translation vectors are predicted for the Cα atoms, while for the side-chain frames, the translation vectors are predicted for the carbon atoms immediately following each of the side-chain torsion angles ($\chi_{1-4}$). Given the predicted translation vectors and the full set of backbone and side-chain torsion angles, the exact 3D structures can be quickly mapped using simple geometric transformations, assuming ideal bond lengths and bond angles.

Along with the pairwise and single representations, the Structure Module takes as input the backbone structure frames, either from those predicted by a previous pass through the network or with translation vectors initialized to the origin and rotations set to the identity if it is the first pass through the network. This iterative process of recycling the output back through the Trunk and Structure Modules allows for continual refinement of predicted structures and enables the network to achieve very high accuracy. The Structure Module augments each of the attention queries, keys, and values from the transformer architecture with 3D points produced in the local frame of each residue, which allows the final values to be invariant to global rotations and translations. Another feature of the Structure Module is that it does not constrain the peptide bonds and allows the network to break the protein chain constraints in order to refine all parts of the protein structure simultaneously. In addition to the backbone frames, the side-chain frames and the estimated residue-level errors are predicted using small per-residue networks based on the final activations at the end of the network. This novel network architecture construction enables an efficient end-to-end training protocol, which is built on the comparison between the predicted and true atom positions, and achieved exceptional structure modeling accuracy, as will be discussed in the next section.

## Impact of deep learning on structure modeling accuracy

The community-wide CASP experiments provide an objective method to benchmark the state of the art of protein structure prediction with different categories for both tertiary structure modeling and contact prediction. As such, the progress made in the field should be best highlighted by reviewing the results of the most recent CASP experiment (CASP14), which took place in 2020, in comparison to previous CASP experiments. Starting from CASP7, the proteins modeled during CASP have been classified as TBM, TBM-easy, TBM-hard, FM/TBM, or FM depending on the availability and quality of PDB templates for each target, where TBM-easy targets have readily identifiable, high-quality templates and FM targets typically lack homologous templates in the PDB. For the purpose of our analysis in the following sections, TBM, TBM-easy, TBM-hard, and FM/TBM targets are all regarded as TBM targets, and FM targets are treated separately. In CASP, predictions are produced by both server groups and human groups. Server groups must deploy fully automated pipelines and submit their result within 72 h while remaining completely blinded to other groups’ predictions. On the other hand, Human groups are given 2 weeks for most modeling targets to allow for more human intervention, such as drawing insights from the final submission of server groups. Due to the longer computational times provided and the full knowledge of the results of all server groups, human groups often perform better than server groups using similar algorithms. In Table 2, we list the top performing groups in CASP14 with available online servers or source code so that readers may access their resources.

**Table 2:** Summary of the current state-of-the-art structure prediction methods, including their results in the most recent CASP experiment and their web server URL addresses

Method	CASP14 group name	CASP14 results^a^	Description; URL address
D-I-TASSER	Zhang-Server	First Place Server	Template and deep learning distance/orientation/hydrogen bond network-guided folding; https://zhanglab.dcmb.med.umich.edu/D-I-TASSER/
D-QUARK	QUARK	Second Place Server	Deep learning distance/orientation-guided folding; https://zhanglab.dcmb.med.umich.edu/D-QUARK/
AlphaFold2	AlphaFold2	First Place Human Group	End-to-end deep learning-based model prediction; https://github.com/deepmind/
Rosetta	BAKER	Second Place Human Group	Deep learning distance/orientation-guided folding; Robetta Server: https://robetta.bakerlab.org trRosetta Server: https://yanglab.nankai.edu.cn/trRosetta/

a Methods in CASP are divided into server and human groups. Predictions by server groups are fully automated, whereas those by human groups do not have to be.

### Improving contact prediction accuracy using deep learning

The most notable development in recent CASP experiments is the employment of deep learning strategies, in particular the incorporation of contact and distance maps derived from deep learning into structure prediction programs. CASP has not introduced a category for distance map prediction, but it does have a contact map prediction competition. In addition, contact maps can be derived from distance maps by collapsing them into two bins.

Figure 5*A* highlights the progress made in contact prediction accuracy over the previous four CASP experiments. The figure shows that a dramatic increase in contact prediction accuracy can be seen not only for the top predictors, but also across the board. The average precision of the top *L*/5 long-range contacts, where *L* is the protein length and long-range indicates contacts between positions separated by at least 24 residues, for the best predictor increased from 26.7% in CASP11 to 74.4% in CASP13. Therefore, remarkably, from 2014 to 2018, the contact prediction precision nearly tripled as a result of the development of contact predictors that utilize deep residual neural networks starting from coevolutionary data.

While in CASP14 the average precision of the best predictor was 75.1%, which was similar to the best CASP13 predictor, the dependency on the number of effective sequences in an MSA shown in Figure 5*B* significantly decreased from CASP13 to CASP14. This is critical progress as most deep learning-based contact/distance map prediction methods utilize coevolutionary features, which require MSAs with many sequences in order to reliably ascertain the coevolutionary couplings between each position. This can be clearly seen in the results for CASP11 and CASP12 in Figure 5*B*, where the contact prediction accuracy was very low when few sequences were available. Thus, the small increase in accuracy between CASP13 and CASP14 may be attributed to the presence of more difficult modeling targets, where deep learning clearly decreases the number of sequences necessary to successfully predict residues that are in contact with each other. Furthermore, a marked increase in accuracy can be observed for the remainder of the predictors in CASP14 as compared with CASP13.

### Improving tertiary structure modeling using deep learning

Traditionally, the most reliable method for predicting protein structures has been to use TBM approaches, which rely on identifying homologous templates from the PDB library in order to model a target sequence. Thus, the accuracy of such TBM approaches is highly dependent on the ability to identify high-quality templates from the PDB library, where the modeling accuracy sharply declines when only low-quality templates are able to be identified. In theory, FM approaches are not limited by the availability of templates in the PDB library, but they have traditionally been outperformed by TBM methods, especially for targets with readily identifiable templates in the PDB. Nevertheless, the incorporation of predicted pairwise restraints from deep learning, and more recently end-to-end learning, into FM approaches has shown promise to close the accuracy gap between TBM and FM methods.

**Figure 6:**
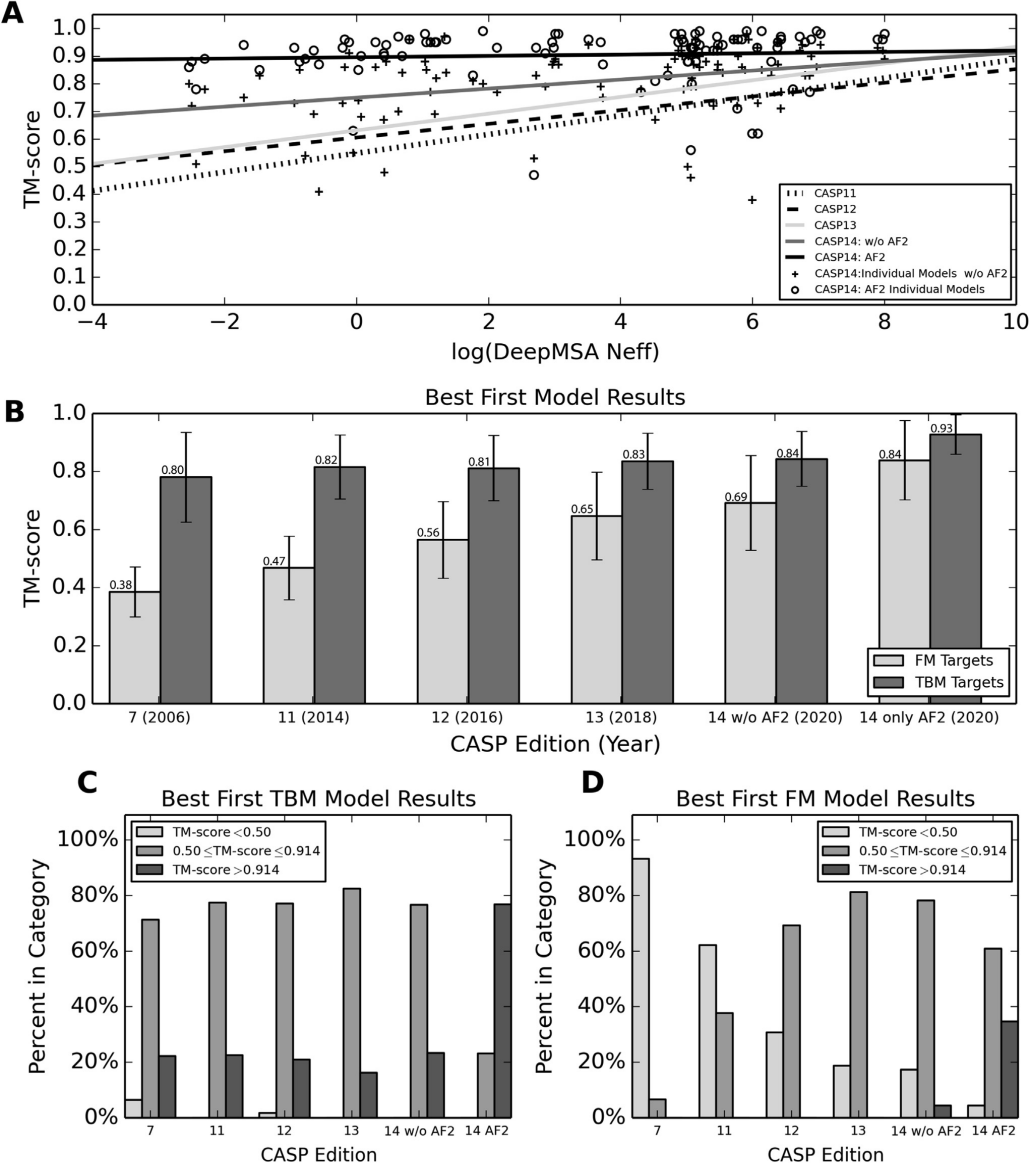
**Summary of structure prediction results in the recent CASP experiments.***A*, relationship between the best TM score of the first submitted model and the Neff value of the MSA generated by the DeepMSA program (184). *B*, mean TM score of the best first TBM and FM models submitted in the corresponding CASP competitions. *C*, results for the best first TBM models (including TBM, TBM-easy, TBMA-hard, and FM/TBM) submitted by any group in CASP7/11 to 14, where the models are categorized into one of three categories based on their TM scores: [0, 0.5), [0.5, 0.914], (0.914, 1.0]. *D*, results for the best first FM models submitted by any group in CASP7/11 to 14, where the models are categorized into one of three categories based on their TM scores: [0, 0.5), [0.5, 0.914], (0.914, 1.0].

Figure 6*B* shows the results from previous CASP experiments as well as the most recent experiment on FM and TBM targets in terms of the mean TM score of the best first submitted model for each target. Here, TM score is a sequence length-independent metric that ranges from [0, 1], where a score >0.5 indicates that the predicted and native structure shares the same global topology and a score >0.914 may be used as a cutoff for low-to-medium resolution experimental accuracy (194, 195). From the plot, it can be seen that the gap in modeling accuracy between FM and TBM targets has narrowed as the field has advanced. In particular, the improvement in FM model quality may be attributed to the use of deep learning restraints and end-to-end learning, as in the absence of suitable template structures, deep learning may be used to guide the structure assembly simulations. In CASP7, the mean TM score for FM and TBM targets was 0.38 and 0.80, respectively, which resulted in a TM-score gap of 0.42. CASP11 saw the gap slightly narrow to 0.35 with a mean TM score equal to 0.47 and 0.82 for FM and TBM targets, respectively. However, as seen in Figure 5*A*, the contact map prediction accuracy was not high enough to make a profound impact on modeling accuracy. As the contact map prediction accuracy improved in CASP12, the FM modeling accuracy also improved to 0.56, while the TBM accuracy remained at 0.81.

As noted in the previous section, CASP13 witnessed a remarkable improvement in contact prediction accuracy due to the use of deep ResNets. Correspondingly, the FM modeling accuracy improved to a mean TM score of 0.65 and the TBM accuracy improved slightly to 0.83, which narrowed the TM-score gap to 0.18. For CASP14, we considered two groups separately: CASP14 without AlphaFold2 models and CASP14 only considering the AlphaFold2 models. Besides AlphaFold2, the top predictors in CASP14 utilized pairwise restraints such as interresidue distances, orientations, and hydrogen bond networks to guide their folding simulations. Therefore, without considering AlphaFold2, the FM modeling accuracy increased to 0.69 and the TBM modeling accuracy increased to 0.84, corresponding to a mean TM-score gap of 0.15. It is interesting to note that there were considerably more FM targets in CASP14 that had few sequence homologs (low Neff, Fig. 5*B*) than in CASP13. This indicates that the CASP14 FM targets were in general more difficult than the CASP13 FM targets, so the results may have been more significant when tested on a similar subset of proteins. Lastly, AlphaFold2 by itself was able to produce extremely accurate predictions with a mean TM score of 0.84 for FM targets and 0.93 for TBM targets (corresponding to a TM-score gap of 0.09). Thus, AlphaFold2 was able to generate FM predictions with accuracies comparable to TBM models generated by other groups, and their models for TBM targets had an average accuracy comparable to low-to-medium resolution experimental structures. Interestingly, we still see a gap in CASP14, albeit a significantly smaller one than observed in previous CASPs, between the modeling accuracy of FM and TBM targets, with a *p*-value of 8.9E-5 as determined by a two-tailed Student’s *t* test. Nevertheless, the 50-year-old gap between FM and TBM modeling accuracies has largely been bridged through the use of deep learning, where solving the protein structure prediction problem may no longer rely on direct identification of global templates from the PDB library.

Deep learning has not only largely closed the gap between the accuracy of TBM and FM approaches, it has also drastically improved the modeling accuracy for targets with few homologous sequences. From Figure 5*B*, we can see the reliance on the number of sequences in an MSA dramatically decreased from CASP12 to CASP13 with the use of deep ResNets, which in turn improved the modeling accuracy for low Neff targets. In CASP14, AlphaFold2’s final model quality was almost completely independent of the MSA Neff value, which is a truly remarkable achievement (Fig. 6*A*). From Figure 6*C*, we can also see a marked increase in the number of models produced with experimental accuracy (when considering a cutoff TM score of 0.914). In previous CASP experiments, no FM targets could be folded with such high accuracy, but in CASP14, AlphaFold2 was able to fold more than 1/3 of the FM targets with experimental accuracy and almost 80% of the TBM targets.

As a note, although the gap between TBM and FM accuracies has been largely reduced and most of the structure prediction studies have focused on distant-homology modeling, in which close homologous templates must be excluded to facilitate benchmark testing and comparisons with other methods, both traditional TBM/FM and modern deep learning methods rely essentially on the experimentally solved structures and are therefore impacted by the increase in the number of structures in the PDB. First, the newly solved experimental structures can provide close homologous templates for more sequences to facilitate high-resolution TBM structure modeling. Second, a larger set of PDB structures contain more comprehensive fold types, which can facilitate the development of more robust knowledge-based statistical force fields and machine learning models for FM. In this context, despite the significant progress in structural bioinformatics, the effort from the experimental structural biology community has been and will continue to be a fundamental driving force to further improve the accuracy of computational protein structure prediction.

## Conclusion and future directions

The prediction of protein structures starting from amino acid sequences has remained an outstanding problem in structural biology since Anfisen first demonstrated that the information encoded in a protein sequence determines its structure. Now more than ever, there is an urgent need to develop high-accuracy protein structure prediction algorithms, as advancements in high-throughput sequencing technology have greatly exacerbated the gap between the number of known protein sequences and the number of experimentally determined structures. Until recently, the most reliable approach for solving the protein structure prediction problem has been to identify and refine the structural frameworks of templates detected from the PDB. This template-based modeling approach works well when homologous templates can be readily detected, but the accuracy sharply declines when only distantly homologous templates exist for a target. Furthermore, traditional template-free modeling approaches have only been able to consistently and accurately fold relatively small non-beta proteins due to compounding inadequacies in the energy functions and conformational sampling techniques used by such approaches. Until recently, for some time, progress in the field has been slow and only incremental gains have been achieved. Nevertheless, the most recent advancements in deep learning-based restraint prediction and end-to-end folding have revolutionized the field of protein structure prediction, greatly improving its accuracy and the ability to fold proteins that lack homologous templates in the PDB. Moreover, the results of the most recent CASP experiment best highlight the progress made in the field, where the use of end-to-end learning and attention-based networks by AlphaFold2 has largely solved the protein structure prediction problem at the domain level.

**Figure 7:**
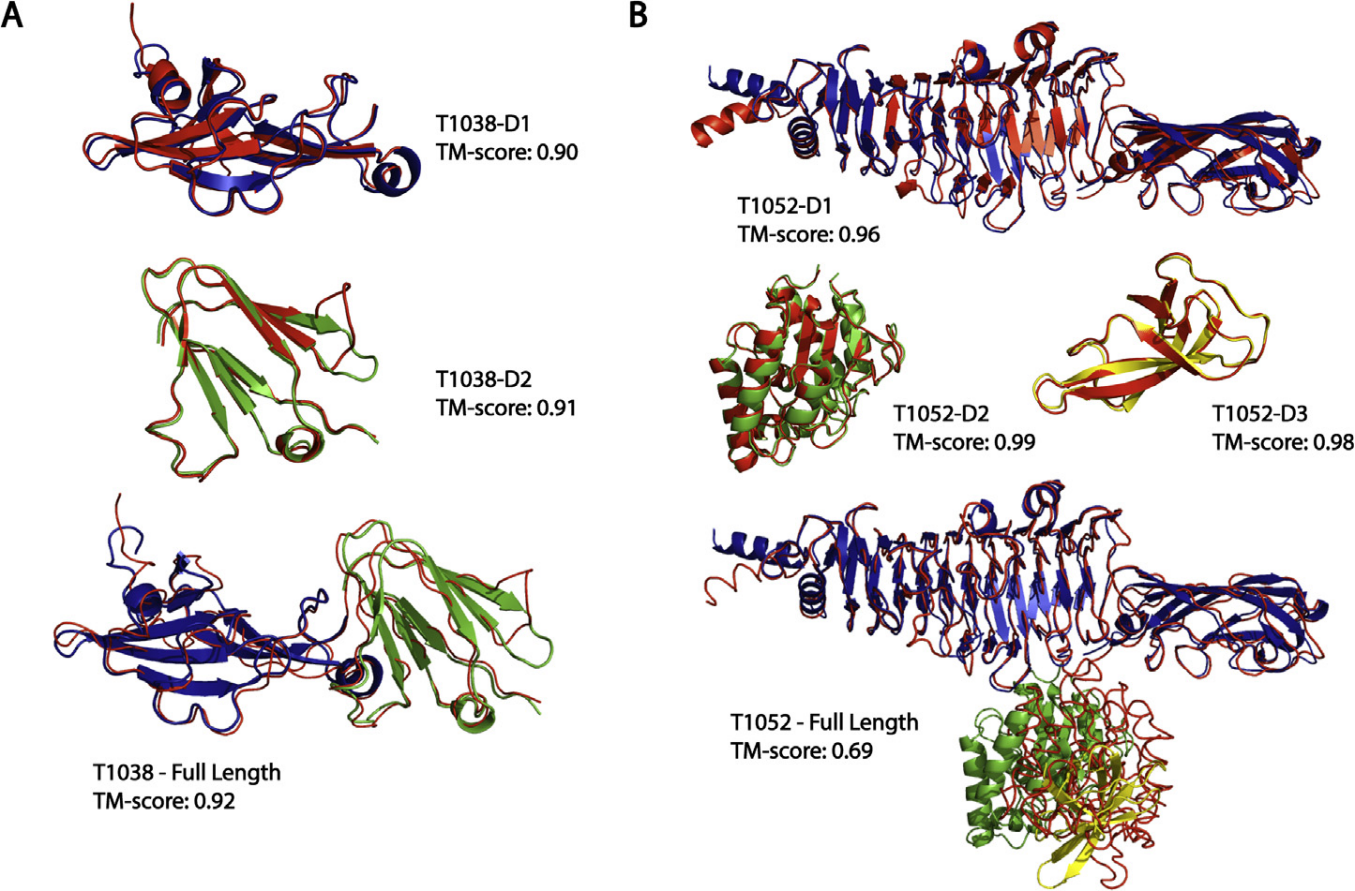
**Representative examples of AlphaFold2 on multidomain protein structures in CASP14.** The experimental structures are shown in *red* cartoons, while the predicted models are shown in different colors for different domains. *A*, modeling results for T1038, where AlphaFold2 achieved excellent performance on both the domain-level and full-length models. *B*, modeling results for T1052, where the domain-level models achieved an extremely high accuracy, but the full-length assembled structure had incorrect domain orientations.

Despite the impressive achievement, there still exists some room for improvement. For example, while the gap between FM and TBM modeling accuracies has been dramatically reduced, there still exists some disparity between the two types of targets as roughly 80% of the TBM targets could be folded with experimental accuracy by AlphaFold2, while only 35% of the FM targets achieved the same accuracy. Moreover, CASP assesses the performance of predictors on single-domain targets, thus AlphaFold2’s performance on more complex multiple domain targets remains unknown, although individual examples with remarkable modeling accuracy have been witnessed for multidomain protein targets in CASP14. Table 3 lists the TM scores for 12 CASP14 multidomain proteins for which at least one domain belonged to an FM target and the full-chain structures were released. For these examples, the average TM score of the assembled full-length proteins was 0.82 compared with 0.91 for each individual domain model. In Figure 7, we list two representative models from targets T1038 and T1052 produced by AlphaFold2. T1038 was composed of two domains that were both FM targets, where the TM scores for the models constructed by AlphaFold2 for domains 1 and 2 were 0.90 and 0.91, respectively, and the full-length model achieved a TM score of 0.92. Thus, AlphaFold2 was able to generate highly accurate domain-level and full-length models. Of particular interest, the next best group only achieved a TM score of 0.43 for the full-length model and a TM score of 0.48 and 0.66 for domains 1 and 2, respectively. This case illustrates the exceptional performance of AlphaFold2 at generating models, particularly for targets that could not be folded by any other group. For T1052, however, the full-length model was significantly worse than the individual domain models. Here, target T1052 was composed of three domains, where AlphaFold2 modeled each individual domain with very high accuracy, achieving TM scores of 0.96, 0.99, and 0.98 for domains 1 to 3, respectively; however, the full-length model was much worse with a TM score of 0.69. Thus, although AlphaFold2 achieved remarkable success in modeling multidomain structures, the full-length modeling accuracy appears to be worse on average than that for the constituent domains; this shows the necessity of further effort on interdomain orientation modeling for protein structure prediction.

**Table 3:** Summary of AlphaFold2’s modeling performance on CASP14 multidomain targets and each constituent domain

Target	Domain (length)	TM-score
T1038	Full Length (L = 190)	0.92
	Domain 1 (L = 114)	0.90
	Domain 2 (L = 76)	0.91
T1047s2	Full Length (L = 346)	0.77
	Domain 1 (L = 147)	0.96
	Domain 2 (L = 83)	0.93
	Domain 3 (L = 116)	0.62
T1052	Full Length (L = 832)	0.69
	Domain 1 (L = 539)	0.96
	Domain 2 (L = 213)	0.99
	Domain 3 (L = 80)	0.98
T1053	Full Length (L = 576)	0.97
	Domain 1 (L = 405)	0.99
	Domain 2 (L = 171)	0.95
T1058	Full Length (L = 382)	0.96
	Domain 1 (L = 221)	0.94
	Domain 2 (L = 161)	0.96
T1061	Full Length (L = 838)	0.77
	Domain 1 (L = 464)	0.93
	Domain 2 (L = 271)	0.81
	Domain 3 (L = 103)	0.95
T1070	Full Length (L = 321)	0.49
	Domain 1 (L = 76)	0.62
	Domain 2 (L = 101)	0.97
	Domain 3 (L = 76)	0.78
	Domain 4 (L = 68)	0.95
T1085	Full Length (L = 406)	0.94
	Domain 1 (L = 167)	0.95
	Domain 2 (L = 182)	0.98
	Domain 3 (L = 57)	0.83
T1086	Full Length (L = 381)	0.94
	Domain 1 (L = 193)	0.96
	Domain 2 (L = 188)	0.96
T1093	Full Length (L = 629)	0.94
	Domain 1 (L = 141)	0.88
	Domain 2 (L = 382)	0.95
	Domain 3 (L = 106)	0.93
T1094	Full Length (L = 484)	0.91
	Domain 1 (L = 277)	0.87
	Domain 2 (L = 207)	0.96
T1096	Full Length (L = 426)	0.56
	Domain 1 (L = 255)	0.94
	Domain 2 (L = 171)	0.85
Average	Full Length (L = 484.3)	0.82
	Domains (L = 187.5)	0.91

Additionally, as many proteins perform their function through interactions with other proteins in a cell, the extension of end-to-end learning to the prediction of protein complex structures and assemblies remains an open problem. Another interesting dilemma to consider is that AlphaFold2 was trained on experimentally solved structures, where the most prominent method for structure determination is X-ray crystallography. Since X-ray crystallography involves crystal formation, the conformation of the protein may not actually be reflective of the biological conformation. Therefore, the extension of deep learning to elucidate protein folding dynamics and the ability to more accurately represent the set of biological conformations adopted by a protein molecule is an interesting future direction. Furthermore, even though CASP represents a rigorous method to validate a protein structure prediction approach, more large-scale tests are still needed. In particular, although only a very small correlation was observed between the final model quality by AlphaFold2 and the MSA depth obtained from a third-party MSA collection program (184), a more systematic study should identify if there is indeed some effect of MSA depth on model performance. This is especially important for targets with very few sequence homologs. Given all these considerations, more work must be done before a complete solution to the protein structure prediction problem can be confidently asserted. Nevertheless, the rapid progress witnessed within the past few years alone provides hope that the complete protein structure prediction problem may be solved using deep learning within the foreseeable future, where predictions may consistently achieve accuracies that rival and even exceed experimental methods.

## Conflict of interest

The authors declare that they have no conflicts of interest with the contents of this article.
